# Genomics for Ruminants in Developing Countries: From Principles to Practice

**DOI:** 10.3389/fgene.2018.00251

**Published:** 2018-07-13

**Authors:** Vincent Ducrocq, Denis Laloe, Marimuthu Swaminathan, Xavier Rognon, Michèle Tixier-Boichard, Tatiana Zerjal

**Affiliations:** ^1^Génétique Animale et Biologie Intégrative, Institut National de la Recherche Agronomique, AgroParisTech, Université Paris-Saclay, Jouy-en-Josas, France; ^2^BAIF Development Research Foundation, Pune, India

**Keywords:** genomic selection, dairy cattle, India, genetic resources, adaptation, NGO, capacity building

## Abstract

Using genomic information, local ruminant populations can be better characterized and compared to selected ones. Genetic relationships between animals can be established even without systematic pedigree recording, provided a budget is available for genotyping. Genomic selection (GS) can rely on a subset of the total population and does not require a costly national infrastructure, e.g., based on progeny testing. Yet, the use of genomic tools for animal breeding in developing countries is still limited. We identify three main reasons for this: (i) the instruments for cheap recording of phenotypes and data management are still limiting. (ii) many developing countries are recurrently exposed to unfavorable conditions (heat, diseases, poor nutrition) requiring special attention to fitness traits, (iii) a high level of expertise in quantitative genetics, modeling, and data manipulation is needed to perform genomic analyses. Yet, the potential outcomes go much beyond genetic improvements and can improve the resilience of the whole farming system. They include a better management of genetic diversity of local populations, a more balanced genetic progress and the possibility to unravel the genetic basis of adaptation of local breeds through whole genome approaches. A GS program being developed by BAIF, a large Indian NGO, is analyzed as a pilot case. It relies on the creation of a female reference population of *Bos indicus* and crossbreds, recorded with modern technology (e.g., smartphones) to collect performances at low cost in tiny herds on production and fertility. Finally, recommendations for the implementation of GS in developing countries are proposed.

## Introduction

The demand for animal products in developing countries is growing at an unprecedented rate due to a combination of factors, including steady population growth, diffuse urbanization and rising levels of family incomes (Steinfeld et al., 2006; Rothschild and Plastow, 2014). Environmental constraints, at present and expected to occur with climate change, are particularly severe in developing countries and require a new balance between adaptation and productivity, as compared to breeding programs in temperate countries where environment is usually better controlled Consequently, the two main features to consider for animal breeding in developing countries are (i) the need for more balanced selection objectives, and (ii) the interest of crossbred or composite populations, to combine adaptation and production ability in various environments (Rege et al., 2011).

The aim of this paper is to analyze, through a pilot case, how genomics can be used to set up novel breeding programs matching the specific needs of developing countries.

## Part 1: Current Contribution of Genomic Information to Animal Breeding in Developing Countries

### New Knowledge Brought by Genomics

Genomics has already greatly improved our knowledge of animal genetic resources in developing countries. Many studies were initiated with microsatellite markers and are now extended to high density (HD) SNP markers sets and whole genome sequencing, as illustrated in goats (Ajmone-Marsan et al., 2014). All studies regularly observed higher genetic diversity in local populations of developing countries for all livestock species (Groeneveld et al., 2010), including cattle (Kim et al., 2017). These studies also made possible the identification of introgression events from exotic breeds and showed that original local populations were still present, thus constituting a genetic resource for animal breeding in developing countries. Analysis of HD SNP data sets on local populations could detect selection signatures associated with adaptation to harsh conditions, mainly those of tropical countries exhibiting hot conditions and pathogens pressure (Gautier et al., 2009; Perez O’Brien et al., 2014; Taye et al., 2017). Thus, selection objectives for breeding in developing countries should not be directly copied from what is applied in temperate countries, even for production systems when environmental conditions can be controlled.

Although molecular data significantly improve our knowledge of animal genetic resources in developing countries, they do not benefit yet to breeding programs in these countries. Classical selection requires an elaborate multi-step breeding program, including pedigree recording, phenotyping and breeding value estimation, which is particularly difficult to organize in a developing country. Could molecular data change the picture?

### Making Use of Molecular Data by Genomic Selection in Ruminants

Genomic selection has completely changed the organization of selection in dairy cattle (Boichard et al., 2016). The possibility of using a whole-genome set of markers to improve the accuracy of breeding value prediction was first described by Meuwissen et al. (2001). It consists in using a set of genotyped and phenotyped animals, called the reference population, to estimate marker-phenotype association which makes possible to predict the breeding value of a calf without the need for progeny testing (PT), thereby reducing generation interval and cost of testing. Key factors of success are the size and the design of the reference population and the access to an informative SNP chip suited to the population (Boichard et al., 2016). Moreover, at least in theory, a higher number of bulls can be proposed to farmers and the management of genetic variability within a breed can be better monitored. Here, genotypes can replace pedigree recording and the set-up of a breeding program may start on a new basis, as compared to mandatory pedigree recording, often a limiting factor in developing countries.

Such a concept was tested on a real data set of 1,013 dairy cows in Kenya, which exhibited various degrees of crossbreeding with exotic breeds (Brown et al., 2016). A principal component analysis based on SNP data showed that individuals could be clustered in three groups according to the proportion of exotic breeds, with a reference and a validation data set for each group. The accuracy of genomic prediction (measured as the correlation between milk yield deviation and genomic breeding value) ranged from 0.32 to 0.41 with GBLUP and from 0.28 to 0.39 with BayesC with no significant difference of performance between the two methods. Considering that pedigree recording was totally missing, this approach opens the way to the set-up of a breeding program but limitations were identified regarding the cost of genotyping and the collection of more phenotypic data.

## Part 2: Implementing Genomic Selection in Developing Countries: a Case Study in India

In this section, we use the example of BAIF Development Research Foundation^1^, a large Indian NGO, as a pilot case to describe examples of constraints and challenges faced when developing a large-scale dairy cattle breeding program in tropical conditions. For 50 years, BAIF’s main mission has been to provide sustainable livelihood to Indian smallholder dairy farmers, in particular by promoting genetic improvement of “non-descript” low yielding cattle (and also buffaloes, but they are not considered here). This is carried out through artificial insemination (AI) using frozen semen technology.

### Characteristics of BAIF’s Selection Program

BAIF was one of the pioneer organizations to introduce AI crossbreeding of cows with “exotic” *Bos taurus* bulls (Holstein and Jersey) in India, which now contributes to more than 50% of the country’s milk production. It expanded to such a point that in 2016, BAIF’s semen stations produced 12.5 million doses of semen from: (i) purebred “exotic” Holstein and Jersey bulls born in BAIF’s bull dam nucleus herd which was created about 40 years ago from heifers imported from Canada and Denmark; (ii) purebred indigenous *Bos indicus* bulls, mainly of Gir and Sahiwal breeds which have a greater milk production potential, but also of other local (draft) breeds for the purpose of genetic resources conservation; (iii) crossbred bulls exhibiting a range of 50–75% exotic blood.

About 4,500 BAIF AI technicians, each covering 12–15 villages, provide AI at the doorsteps of poor families as well as basic guidance on animal nutrition, health, and management. BAIF is currently serving over four million rural households in 16 states all over India (roughly excluding the extreme South, North, and East states) with very diverse agro-climatic conditions, in terms of temperature, water resources, farming systems and production constraints. The most striking common feature is the very small herd size (<2).

### Initial Selection Practices

Since 1994, BAIF has been part of a field PT program run by the Indian Council of Agricultural Research (ICAR). Under this program, phenotype recording is only on milk yield and is quite costly given the herd size: each cow is recorded every 14 days, in order to obtain an accurate lactation yield. Recording takes place mostly in Maharashtra villages with a long experience with BAIF. Unfortunately, up to 70% of the records are lost, mainly because of unknown sire, animal identification errors or transcription mismatches when entering information in the database. As a result, only a small fraction of all BAIF Holstein and Holstein crossbred bulls have been progeny tested. The best PT bulls have been used as sires of sons and in the most productive villages, which are also the ones that have practiced crossbreeding for the longest time. In practice, non-progeny tested bulls as well as bulls waiting for PT results have to be used continuously (no lay-off period). Therefore, PT, which has made dairy cattle selection so efficient in many countries, is just costly, inappropriate, and quite ineffective under Indian conditions. Clearly, the main bottleneck for a more ambitious bull selection based on PT was, and still is, the implementation of low cost, large scale recording in tiny herds.

### Selection Objectives

There are other important limitations with the BAIF’s current PT program: it concentrates mainly on the recording and selection of just one trait: milk production, despite the fact that in India, milk price highly depends on fat content. Also, the huge heterogeneity of agro-climatic conditions generates large genotype × environment interactions, which have to be accounted for in selection at different levels (choice of breed, of fraction of exotic blood for crossbred bulls, of individual bulls). Selecting only on production traits strongly favors animals with (too) high levels of exotic origin and adaptation to the local conditions can be rapidly lost.

Cow longevity is an obvious trait reflecting adaptation, but is not pertinent in India where slaughter of unproductive cows is not permitted. Considering morphological traits such as good udders, feet, and legs can help but is not enough. The infrastructure for large scale recording of health traits, in particular resistance to mastitis, does not exist yet. A more accessible trait to collect which can be considered as a proxy for general adaptation may be fertility: an unfit or unhealthy cow is less likely to be fertile. At BAIF, AI information is of good quality, with a systematic pregnancy diagnosis two months after each insemination. Combined with proper tagging, good AI and calving records are also important prerequisites to ensure correct pedigree information required in genetic evaluations. Another frequently overlooked aspect to keep in mind in bull selection in India are the farmer’s expectations and beliefs (coat color or pattern, shape of horns, or ears, etc.) for good acceptance in the field.

### Low Cost Collection of Phenotypes

The possibility to collect field data at BAIF on a much larger scale was investigated through a project (the “Godhan project”) sponsored by the Bill and Melinda Gates Foundation (BMGF): 170 AI technicians were equipped with multi-component software, installed first on dedicated “data loggers” and later on mobile phones. Originally developed to follow the economic and social status of BAIF farmers over time, the software was extended to include technical data. Soon, hundreds of thousands of good quality records were gathered, in particular on fertility, avoiding the error-prone process of data entry and validation (Potdar et al., 2017). It was originally planned to also ask the AI technician to directly collect milk production data from the farmers but this appeared to be difficult, probably because the farmers – as well as the AI technicians – were not motivated enough with incentives and above all, proper feedback. Hence, large scale, low cost milk sample collection and analysis (for fat and protein content or for somatic cell counts) remain an issue.

### Toward Genomic Selection

Even with the low cost of large scale performance recording, generating a group of progeny tested bulls of reasonable size to start genomic evaluation is a long and complex process, in particular because of the very limited population with pedigree information: an incompressible preliminary period is necessary before tagged daughters from known sires start being recorded.

Most of the constraints and challenges indicated above lead to the notion of promoting the development of female reference populations (FRPs), which replace the requirement for a large-scale recording infrastructure by a more realistic collection of phenotypes from a set of genotyped cows. These phenotypes should cover the traits identified in the selection objective and come from herds with carefully documented environmental and management characteristics, hence offering the possibility to actually measure G × E interactions on all traits. Absence of known pedigree relationships is overcome by using genomic information, the cost of which cannot be covered by small farmers. Since the constitution of FRP requires strong and long-term financial and technical support from governmental or international institutions, the BAIF project benefits from an important BMGF sponsoring for 5 years, where more than 15,000 pure and crossbred indigenous cows, mainly coming from six very diverse Indian states, are phenotyped, and a substantial portion of them are being genotyped.

### Technology and Infrastructure

The commercially available medium- or low-density SNP chips were primarily designed for *Bos taurus* cattle. For *Bos indicus* and crossbred animals at BAIF, these chips are suboptimal because a substantial number of SNP have a very low minimum allele frequency, a low heterozygosity or are fixed (Strucken et al., 2018). In other words, they are less informative.

In terms of infrastructure, the actual constitution of a completely new reference population is obviously a long and complicated task requiring huge investments in human and material resources and a strong centralized coordination. BAIF could rely on its existing AI technician networks. It must be emphasized that collection of field data requires constant motivation and follow-up at all levels (farmers, technicians, supervisors). At a central level, the design and maintenance of a high quality database is also essential.

A critical step toward genomic selection is the data analysis and the development of prediction equations. They require a high level of expertise in quantitative genetics, modeling and data manipulation. A potentially overlooked difficulty is the choice of a proper genetic evaluation model, actually reflecting the factors contributing to the observed variability of performances. Developing a sophisticated genomic evaluation based on a simplistic genetic evaluation is strongly counterproductive. At BAIF, technical support from University of New England, Australia, and INRA, France, is available for this applied research work.

A final challenge is transforming research developments and results into a continual data stream and a sustainable genomic evaluation procedure that will routinely provide genomic breeding values of bull and bull dam candidates to selection.

## Part 3: Recommendations for Application of Genomic Tools to Animal Breeding in Developing Countries

### Involving all Stakeholders in the Breeding Program

In 2010, FAO guidelines recommended Community-Based-Breeding-Programs for the management of animal genetic resources. Benefits and limitations of the approach have been previously discussed (Wurzinger et al., 2011). Practical situations analyzed in Bangladesh (Bhuiyan et al., 2017) have led to a set of recommendations underlining the need to : (i) define breeding objectives relevant for the community; (ii) identify the relevant traits to record; (iii) develop inexpensive and easy-to-use devices for phenotype recording, (iv) promote feedback on the program and information exchange. In addition, both studies highlighted the importance of governmental support, with national breeding policies and enabling measures to scale up the programs.

In the case of BAIF, a major leverage is the monitoring of performance of each AI technician as compared to his/her local colleagues. Technicians are equipped with mobile devices that accelerate data collection and improve data quality. This could be a way to provide farmers with rapid feedback on their practices, allowing for improved management of reproduction and nutrition of their cows. Ultimately, the genetic improvement program aims to improve rural livelihoods. The potential long-term outcomes go beyond genetic improvements and can improve the resilience of the whole farming system.

Selection objectives must reflect a real balance between general adaptation, health, and production. This balance has to be carefully addressed because it influences the long term sustainability of farming. Lessons from the BAIF case suggest the need to identify a trait able to represent the expected balance between production and adaptation, considering local constraints and farmers’ preference. Consequently, fertility has been preferred to longevity in India, whereas the latter could be preferred in another context.

### Building the Reference Population

The choice of the breed type and of breed composition in crossbreds should align with the local agro-climatic environment and socio-cultural context, giving priority to animals that cope well with harsh climatic, nutritional, and health conditions. The few examples considering genomic selection tend to favor crossbreeding. Lessons from the BAIF case suggest that a portfolio of purebred or crossbred genotypes is the best answer to the various needs, a strategy which is also described in Bangladesh for ruminants (Bhuiyan et al., 2017). Maintaining various alternatives allows preserving and improving purebred indigenous populations, thus exploiting their specific adaptive features, together with the local production and dissemination of crossbreds.

Whatever the type of animals considered, a very close relationship between the FRP and the on-farm population to be improved is key for the accuracy of genomic prediction. Thus, the FRP must represent the current genetic structure/diversity of the population to be improved, the range of crossbreeding if any, and the range of production conditions (environment and management) because of potential G × E interactions. Thus, a large-scale FRP is required to obtain reliable genomic predictions for populations distributed over a large territory, with little exchange among herds. Two difficulties may arise: (i) inconsistency among agriculture public policies in the case of transboundary populations, (ii) competing initiatives within a country or across countries.

For breeds managed in a large number of small herds, data recording should preferably be standardized among herds, unless appropriate methods are used to account for data heterogeneity (see Methodological Challenges). Data should be analyzed centrally, requiring a full-scale data sharing and a good level of organization.

Cumulative constitution of the FRP is necessary to ensure sustainability of the genomic selection program and a progressive increase in prediction accuracy.

### Methodological Challenges

Lessons from BAIF show that adapting genetic evaluation models (e.g., random regression models) based on test-day records makes possible a better correction for the large environmental changes over the year, and relaxes the requirement of rather strict intervals between consecutive records of a cow (Duclos et al., 2008). Furthermore, the challenge of accounting for local environmental conditions in very small herds could be addressed by including in genetic models a “(group of) village(s) by month” contemporary group as a proxy for herd management.

As aforementioned with the case of BAIF, if the existing SNP chips, developed for *Bos taurus* in developed countries, can allow the genotyping of bovine populations in developing countries, it appears that they may not be fully operational (less informative than expected, especially when using pure *Bos indicus* or *Bos indicus* ×*Bos taurus* crosses). Three alternatives are then possible: (i) accept a loss in accuracy, which may be compensated by a higher number of genotypes with a cheaper *Bos taurus* chip; (ii) create an imputation population of animals genotyped with the HD chip which included some *Bos indicus* breeds, and impute the HD genotype of the whole reference population; (iii) design a new chip fully adapted to *Bos indicus* and crossbred animals. The best option depends on the local conditions. In particular, if the market for a new chip is limited, e.g., when different stakeholders want each to develop a different chip for a same target population, option (iii) may be the less effective one. Such a new chip could be developed as part of a South–South collaboration, involving scientists and breeders from countries concerned by a particular set of breeds to be improved and willing to set up such breeding programs. The sharing of data will lead to a common SNP database from which a suitable chip can be created to be used for marker phenotype association in the FRP. A possibility in the case of BAIF is to envision a transition over time between these options, from (i) + (ii) to (iii). Other alternative options, such as genotyping-by-sequencing have been proposed (Gorjanc et al., 2015), but they should be considered with caution because patents derogation for developing countries may be required.

Exploiting a large number of genotypes at the whole-genome level also opens new possibilities for animal breeding:

– the numerous genotypes being collected for males and females could be used to monitor inbreeding at the genome level and better manage population diversity;

– identification of genomic regions that are common across breeds (with identical directions of allele effects) and that are significantly associated with traits to be improved may help improve across-breed genomic evaluations (Purfield et al., 2015).

### Technological Challenges

Developing countries suffer from deficient tools and infrastructures (Rothschild and Plastow, 2014; Helmy et al., 2016), which limits the use of genomic information in breeding programs.

Reliable marker genotypes require good management of samples for DNA extraction, easy access to experienced genotyping platforms and a proper data base infrastructure. Such structures are often missing or weakly supported for livestock. Therefore, the opportunity of using genotyping or sequencing platforms developed for human genetics should be encouraged to save the cost of establishing expensive dedicated platforms (Glenn, 2011). However, the crucial step for any breeding organization is to master bioinformatics expertise and secure access to computing facilities. As an example, a pan-African network was set up for the “Human Heredity and Health in Africa” initiative^2^, to support access to technologies, facilitate the funding of infrastructures and offer training. In the case of livestock, the interstate Research Center “Centre International de Recherche-Développement sur l’Elevage en zone Subhumide (CIRDES),” based in Burkina Faso and resulting from the partnership between seven West African countries, could play this role in the sub-region.

The lack of sperm production, preservation and dissemination facilities in developing countries has long been reported (Timon, 1993) and remains relevant (Rothschild and Plastow, 2014) in many developing countries. Lessons from BAIF show the benefit from controlling a large-scale infrastructure for AI to fully benefit from the use of genetic information, especially when serving small farmers.

Internet access and easy communication tools (mobile apps) are also very important enhancers, both for the technical supervision of the farms and for the farmers themselves, to facilitate their involvement and appropriation of breeding programs as well as data collection. Thus, internet connections must be effective. Even when such a network exists (Helmy et al., 2016), the lack of stability of the country’s energy infrastructures often causes power cuts and weakens internet reliability (Karikari, 2015).

### Capacity Building

In terms of capacity building, constraints observed in developing countries to enable the implementation of genomics applied to livestock are many and involve human, institutional, logistical and financial aspects (Rothschild and Plastow, 2014; van Marle-Köster et al., 2015; Helmy et al., 2016).

The use of genomic data requires expertise in database development and support, quantitative genetics, and statistical modeling to guarantee accurate and stable genomics analyses.

Yet, setting up genetic improvement programs is worthwhile only when animals’ maintenance feed requirements are covered (McDowell, 1989; Timon and Baber, 1989). To this extent, farmer training courses should provide, on the one hand, basic guidance on animal nutrition, health and management to improve animal welfare and, on the other hand, should explain the requirements in terms of data recording, and raise awareness of pros and cons regarding the choice of a bull or bull type, i.e., purebred or crossbred.

Training programs for scientists and managers of breeding programs are needed in quantitative genetics, genomics and bioinformatics, with access to scientific literature resources (Rothschild and Plastow, 2014; Karikari, 2015; Helmy et al., 2016). South–South and North–South co-operations are to be encouraged to facilitate training.

### Investment

The main drawback of setting up a reference population is the genotyping cost of a large number of animals: the amount of phenotypic information associated with each genotype and available for genomic evaluation is substantially smaller for cows than for progeny tested bulls (Goddard, 2009). This reduction may be even larger in developing countries for two main reasons: a larger equivalent population size of populations with a limited selection history (e.g., for *Bos indicus* cattle) and lower heritability traits due to a much more variable environment and a small herd size.

Using genomic information for the management of genetic variability may be relatively easy, provided that the genotyping cost is affordable, which is not so obvious for small populations. To decrease costs, a multi-breed SNP chip is an option to recommend.

Lessons from BAIF show that a major investor is needed to start a sustainable program, which should be a donor, either a public institution, or a private foundation supporting common goods, such as BMGF. It is of utmost importance to orient these donors toward breeding programs aimed at empowering local communities. Then, long-term operations require a professional and self-supporting organization.

## Conclusion

Genomic selection has the potential to overcome the difficulties encountered by developing countries to implement classical breeding programs where pedigree recording is a pre-requisite. The aim is not to copy breeding programs from temperate countries but to benefit from new methods to better answer the needs of farmers in developing countries. The analysis of a case study provided by BAIF helps to identify the critical factors of success, including: importance of a representative reference population in terms of diversity of genotypes and of environmental conditions; definition of balanced selection objectives and appropriate traits as proxy for adaptation; involvement of farmers and technicians with incentives and quick feedback to them; building local expertise in quantitative genetics and bioinformatics. Challenges consist in accounting for genotype × environment interactions, decreasing genotyping cost by using common tools, getting full advantage of genomic data to combine preservation of genetic diversity with improvement of animal performance, building a sustainable economic model complementary to donor support. A balanced and well monitored use of local and exotic genetic resources is possible. This deserves appropriate public policies allowing for the development of new breeding programs without compromising the importance to preserve local genetic resources.

## Author Contributions

MT-B conceived the paper, drafted part of the paper, and read, discussed, and approved the whole manuscript. VD proposed the case study, drafted part of the paper, and read, discussed, and approved the whole manuscript. MS provided information on the case study, and read and approved the whole manuscript. DL, XR, and TZ drafted parts of the paper, and read, discussed, and approved the whole manuscript.

## Conflict of Interest Statement

The authors declare that the research was conducted in the absence of any commercial or financial relationships that could be construed as a potential conflict of interest.

## Footnotes

1 http://www.baif.org.in

2 https://h3africa.org
